# Effectiveness and Safety of Chinese Medicine Decoctions for Behcet's Disease: A Systematic Review and Meta-Analysis

**DOI:** 10.1155/2021/8202512

**Published:** 2021-07-17

**Authors:** Jingxian Yan, Yi Yan, Andrew Young, Zhiyong Yan, Zhimin Yan

**Affiliations:** ^1^Chinese Medical College, Tianjin University of Traditional Chinese Medicine, Tianjin 301617, China; ^2^Xinglin College, Liaoning University of Traditional Chinese Medicine, Shenyang 110167, China; ^3^Department of Diagnostic Sciences, Arthur Dugoni School of Dentistry, University of the Pacific, Stockton, CA, USA; ^4^Department of Surgery, Handan First Hospital, Handan 056000, China; ^5^Department of Oral Medicine, Peking University School and Hospital of Stomatology, Beijing 100081, China

## Abstract

**Background:**

Behcet's disease (BD) is an autoimmune disease of systemic vasculitis with an unclear pathogenesis. Although western medicines remain the mainstay interventions, effectiveness and safety are significant challenges. Complementary and alternative medicine, including herbal medicine, are gaining more attention. Chinese medicine decoctions, which have been used for centuries, are the most common form of traditional therapies.

**Objective:**

The purpose of the review was to evaluate the effectiveness and safety of Chinese medicine decoctions in the treatment of BD.

**Methods:**

Randomized controlled trials (RCTs) for BD treatment with Chinese medicine decoctions were searched in six electronic databases until March 2021. Primary outcomes were total effective rate, recovery rate, and recurrence rate. Secondary outcomes were clinical feature scores (oral ulcers, eye lesions, genital ulcers, skin lesions, arthropathies, fever, and pathergy reactions) and laboratory index levels (erythrocyte sedimentation rate, C-reactive protein, and immunoglobulin A). The risk of bias was assessed with the Cochrane Handbook, and a meta-analysis was performed with RevMan 5.4.1.

**Results:**

Sixteen RCTs with 924 patients were included in the review. The meta-analysis indicated that Chinese medicine decoctions were effective for BD when compared with control groups for all the primary outcomes and 7/10 of the secondary outcomes. Adverse events were reported in 11 of the 16 RCTs, with the Chinese medicine decoctions possibly having fewer adverse events than western drugs. This review included a range of classical prescriptions. An additional meta-analysis of modified Gancao Xiexin Decoction for BD treatment was conducted. Gancao Xiexin decoction is also discussed as a representative prescription, as well as high-frequency herbs, and warrants further exploration for individualized medicine and pharmacology.

**Conclusion:**

Chinese medicine decoctions have the potential to be effective and safe for treating BD. However, additional well-designed RCTs are needed to confirm the findings because of the unsatisfactory quality of the included studies.

## 1. Introduction

Behcet's disease (BD), also referred to as Behcet's syndrome, is a chronic inflammatory vasculitis with multiple systems involved. It is typically characterized by recurrent canker sores, genital ulcers, ocular lesions, and cutaneous lesions [1]. In most reports, the mean age of onset is 20 to 30 years, and the incidence of the disease is higher in males than females [2]. Although the etiology of BD is not yet clear [3], it is recognized as an autoimmune disease with a genetic predisposition and infection-associated triggering factors, mediated by immune cells, chemokines, and cytokines [4]. The homeostasis perturbation of T cells, especially Th1 and Th17, is now considered to be the main immunological basis of BD pathogenesis [5].

For treatment, corticosteroids, immunomodulatory agents, immunosuppressive agents, and tumor necrosis factor (TNF) alpha inhibitors are commonly used. The main goal of treatment is to prevent the effects of inflammation and inhibit the progression of the disease from reaching the point of target organ damage [6]. However, the long-term disadvantages of western medical treatment include side effects, drug resistance, and relapse after discontinuation of the medication [5]. For example, thalidomide is contraindicated in pregnant women because of teratogenicity [7]. Systemic steroids are associated with a number of adverse events (AEs) and can lead to tolerance [8]. For patients with severe clinical manifestations and intolerance or resistance to standard immunosuppression protocols, TNF blockade might be beneficial, but with relatively high costs [9]. Thus, for certain groups of BD patients, treatment options are limited.

Complementary and alternative medicine, including herbal medicine, are gaining more notice. Herbal medicines have been reported to reduce the risk of disease and improve the body's immunity, such that in the positive role they play in immune regulation in cancer patients [10]. There is also evidence that some of the ingredients in herbal medicines act as anti-inflammatory agents, mimicking flavonoids, steroids, alkaloids, glycosides, polyphenols, curcumins, terpenoids, gamma linolenic acid, phenolic diterpenes, and harpagoside. Compared to synthetic steroid and nonsteroidal anti-inflammatory drugs, they have fewer side effects and lower cost [11].

In traditional Chinese medicine theory, Behcet's disease is similar to Huhuo disease in clinical manifestations and pathogenesis [12]. The presentation of Huhuo was first recorded in Jin-Kui-Yao-Lue (Synopsis of Golden Chamber). In this important reference, the classic treatment prescriptions, including Gancao Xiexin decoction and Chixiaodou Danggui powder, were addressed. After thousands of years of practical application and adjustment, a few more classical prescriptions have been developed that also deserve further attention.

The aim of this systematic review is to provide clinical evidence of Chinese medicine decoctions in the treatment of BD. Although a few systematic reviews have been previously published on herbal medicine for BD, the number of included studies has been limited. Additionally, there is a lack of reviews on Chinese medicine decoctions, even though they are the most common form of traditional therapy. In this review, we conducted a comprehensive search for studies on Chinese medicine decoctions for BD, to provide evidence for using herbal medicine to treat BD, by analyzing effectiveness and safety data. Furthermore, an additional meta-analysis of modified Gancao Xiexin Decoction for BD treatment was also conducted.

## 2. Methods

This review was carried out according to the Preferred Reporting Items for Systematic Reviews and Meta-Analysis (PRISMA): the PRISMA Statement [13].

### 2.1. Databases and Search Strategies

Two reviewing authors (Jingxian Yan and Yi Yan) independently searched 6 electronic databases for randomized controlled trials (RCTs) focusing on Chinese medicine decoctions for treatment of BD: Chinese National Knowledge Infrastructure (CNKI), VIP China Science and Technology Journal Database (VIP), Wanfang Data (Wanfang), Embase, PubMed, and the Cochrane Library until March 31, 2021. The search terms, used individually or combined, included “Behcet's disease,” “Behcet's syndrome,” “BD,” “Behcet disease,” “Behcet syndrome,” “traditional Chinese medicine,” “TCM,” “medicinal herb,” “herbal medicine,” “Chinese medicine,” “traditional medicine,” “herb,” “classical prescription,” and “decoction.” The retrieval strategies used the Cochrane database as an example (see Table S1 in the Supplementary Material). We used hand searching as an adjunctive search method.

### 2.2. Inclusion and Exclusion Criteria

The inclusion criteria were as follows: (a) the studies on the treatment of BD with Chinese medicine decoctions had to be RCTs. (b) Interventions in the experimental group were orally administered Chinese medicine decoctions with or without topical decoctions. (c) The additional intervention (if any) in the experimental group must be the same (including dose, frequency, and route of administration) as the control group. (d) Interventions in the control group could be western drugs (such as thalidomide and prednisone) or placebos. (e) The studies had to report at least one primary outcome and one secondary outcome. (f) The age and gender of patients were not limited. (g) No language restrictions were applied.

The exclusion criteria were as follows: (a) duplicate publications, (b) studies with unavailable full-text, (c) protocols, (d) studies using nondecoction dosage forms (such as capsules, granules, tablets, and substituting tea drinking) or decoctions only by topical application in the experimental group, and (e) intervention combined with herbs in the control group.

### 2.3. Types of Outcome Measures

Primary outcomes were (a) total effective rate: numbers of clinically cured, significant improvement, or improvement/total number; (b) recovery rate: number of clinically cured/total number; (c) recurrence rate. Secondary outcomes were (a) clinical feature scores (oral ulcers, eye lesions, genital ulcers, skin lesions, arthropathy, fever, and pathergy reactions); (b) laboratory index levels (erythrocyte sedimentation rate (ESR), C-reactive protein (CRP], and immunoglobulin A (IgA)). In addition, we documented the AEs mentioned in all the included studies.

### 2.4. Data Extraction

Two reviewing authors (Jingxian Yan and Yi Yan) performed the literature search, study selection, and data collection independently. Extracted data included the title, author, year of publication, type of grouping, type of blinding, sample size, general condition of the patients, intervention of the experimental and control group, treatment course, outcome measures, components of basic decoction, and modification of prescriptions. A third reviewer (Zhiyong Yan or Zhimin Yan) was invited to make an assessment if the two review authors could not reach a consensus.

### 2.5. Risk of Bias Assessment

The risk of bias in the included RCTs was assessed with the RevMan 5.4.1 software (Cochrane Informatics and Knowledge Management Department) and the Cochrane Handbook for Systemic Reviews of Interventions, Version 5.1.0 [14]. The assessment criteria include seven domains: (1) random sequence generation (selection bias), (2) allocation concealment (selection bias), (3) blinding of participants and personnel (performance bias), (4) blinding of outcome assessment (detection bias), (5) incomplete outcome data (attrition bias), (6) selective reporting (reporting bias), and (7) other bias. We described the degree of risk of bias for each domain as “low risk of bias,” “unclear risk of bias,” or “high risk of bias.”

### 2.6. Data Analysis

The efficacy of Chinese medicine decoctions on BD was evaluated using the RevMan 5.4.1 software. For dichotomous data, we chose the Mantel-Haenszel statistical method. Results were expressed as risk ratios (RR) together with the 95% confidence interval (CI) and plotted on a forest plot. For continuous data, we chose the inverse variance statistical method. Results were expressed as mean difference (MD) together with the 95% CI and plotted on a forest plot. The *Q*‐test and *I*^2^ were used to test for heterogeneity of the included studies. When there was no significant heterogeneity (*P* > 0.10, *I*^2^ < 50%), the fixed effect analysis model was used; when there was obvious heterogeneity (*P* < 0.10, *I*^2^ > 50%), the random effects analysis model was used. When the same outcome measure was reported in more than ten RCTs, a funnel plot was used to assess publication bias.

## 3. Results

### 3.1. Search Results

**Figure 1:**
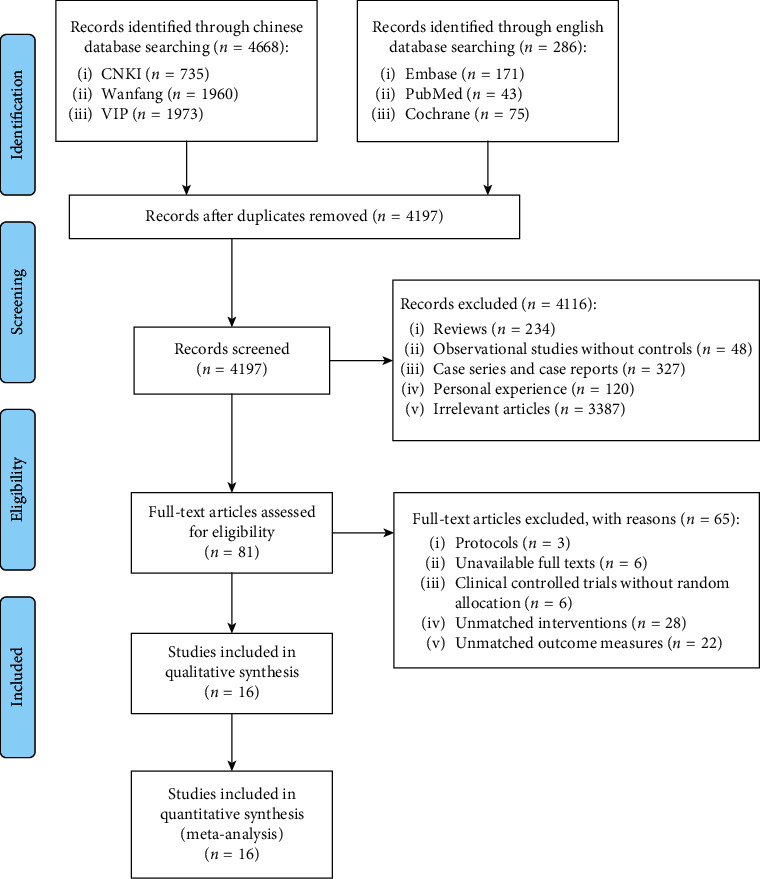
Flow diagram of study selection.

A total of 4957 related articles were obtained by searching the databases. After removing the duplicate articles, 4197 studies remained. After screening the titles and abstracts, 4116 studies were excluded. After full-text reading, 65 studies were excluded, resulting in 16 studies that met the inclusion criteria [15–30]. The process of study selection is shown in Figure 1. No studies that met our requirements were obtained by the hand searching.

### 3.2. Study Characteristics

Seven theses and nine journal articles were included. All the included studies were conducted in China. A total of 924 patients were enrolled in the 16 studies. All 16 included studies were RCTs. Seven RCTs described specific randomization methods including random number tables and statistical software random allocation. Two RCTs explicitly used blinding, and both were single-blind designs. The basic characteristics of the included studies are presented in Table 1. For the control group, western medicine was used in all the included studies. For the experimental group, eight RCTs used only Chinese medicine decoctions, and eight RCTs used Chinese medicine decoctions combined with the same treatment as the control group. Treatment course ranged from two to four months. In each study, the experimental group had the same treatment course as the control group. The interventions and treatment course are presented in Table 2. The outcome measures and AEs are presented in Table 3. All 16 RCTs had a basic decoction in the experimental group, and 12 RCTs revised the prescription according to the patient's condition. There were 102 herbs mentioned in the 16 RCTs. The components of basic decoction and modification of prescriptions are presented in Table S2 in the Supplementary Material.

### 3.3. Risk of Bias

**Figure 2:**
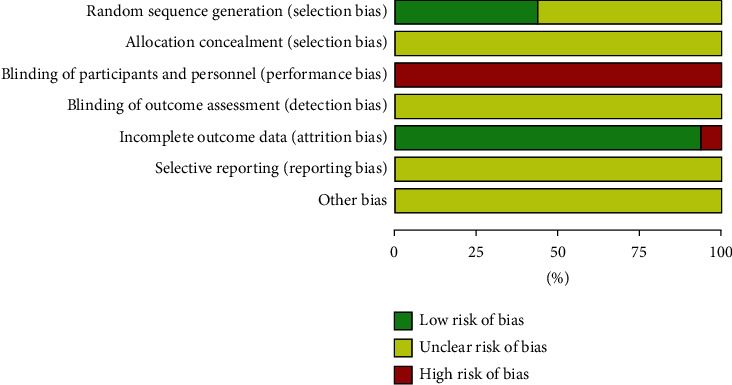
Risk of bias graph: review authors' judgements about each risk of bias item presented as percentages across all included studies.

**Figure 3:**
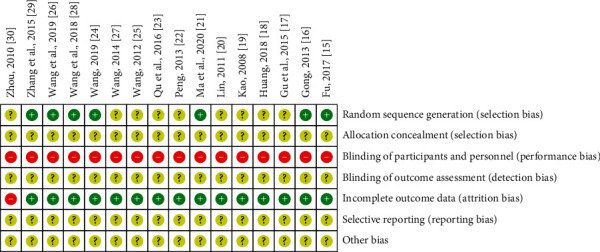
Risk of bias summary: review authors' judgements about each risk of bias item for each included study. +: low risk of bias. −: high risk of bias. ?: unclear risk of bias.

For the “random sequence generation” category, randomization was mentioned in all the included studies, but only seven RCTs presented specific randomization methods. Accordingly, seven RCTs were assessed as low risk, and the remainder was assessed as unclear risk. For “blinding of participants and personnel,” all the included studies were assessed as high risk. For “incomplete outcome data,” one RCT did not report the handling of missing data, and so was assessed as high risk. The other studies were assessed as low risk. For other domains, all the included studies were assessed as unclear risk.  Figure 2 depicts the risk of bias graph.  Figure 3 depicts the risk of bias summary.

### 3.4. Primary Outcomes

#### 3.4.1. Total Effective Rate

**Figure 4:**
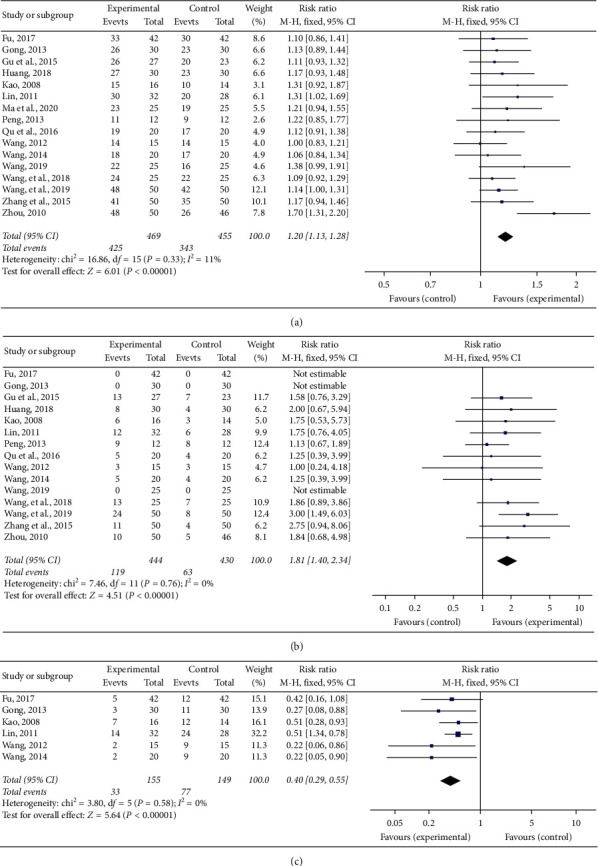
Forest plots of (a) total effective rate, (b) recovery rate, and (c) recurrence rate.

Sixteen RCTs [15–30] compared the total effective rate between the experimental group and the control group. Based on the result of the heterogeneity test (*P*=0.33, *I*^2^ = 11%), the fixed effect analysis model was used to complete the meta-analysis. The meta-analysis showed that the Chinese medicine decoctions had significantly higher total effective rates in the experimental group when compared with the control group (RR = 1.20, 95% CI [1.13, 1.28], *P* < 0.00001; Figure 4(a)).

#### 3.4.2. Recovery Rate

Fifteen RCTs [15–20, 22–30] compared recovery rates between the experimental and control groups. Based on the result of the heterogeneity test (*P*=0.76, *I*^2^ = 0%), the fixed effect analysis model was used to complete the meta-analysis. The meta-analysis showed that the Chinese medicine decoctions had significantly higher recovery rates in the experimental group when compared with the control group (RR = 1.81, 95% CI [1.40, 2.34], *P* < 0.00001; Figure 4(b)).

#### 3.4.3. Recurrence Rate

Six RCTs [15, 16, 19, 20, 25, 27] compared the recurrence rate between experimental and control groups. Based on the result of the heterogeneity test (*P*=0.58, *I*^2^ = 0%), the fixed effect analysis model was used to complete the meta-analysis. The meta-analysis showed that the Chinese medicine decoctions had significantly lower recurrence rates in the experimental group when compared with the control group (RR = 0.40, 95% CI [0.29, 0.55], *P* < 0.00001; Figure 4(c)).

### 3.5. Secondary Outcomes

#### 3.5.1. Oral Ulcer

**Figure 5:**
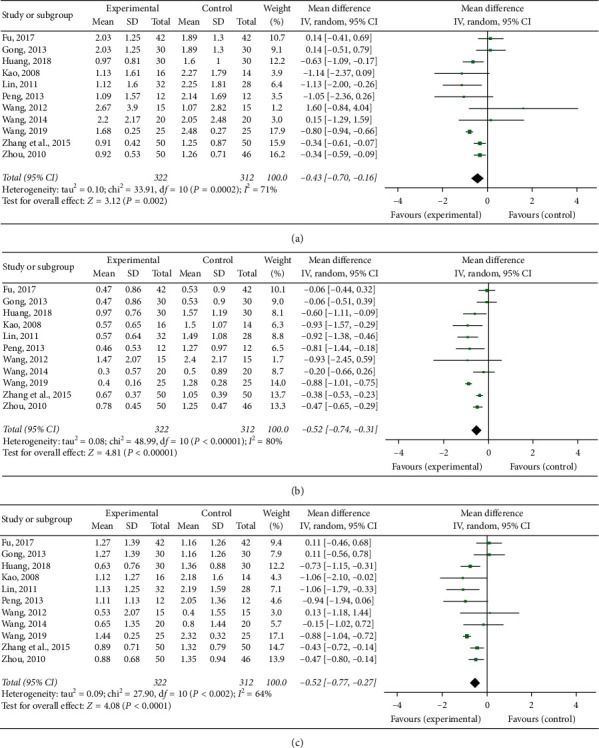
Forest plots of clinical features score: (a) oral ulcer, (b) eye lesion, and (c) genital ulcer.

Eleven RCTs [15, 16, 18–20, 22, 24, 25, 27, 29, 30] quantified oral ulcers. Based on the result of the heterogeneity test (*P*=0.00002, *I*^2^ = 71%), the random effects analysis model was used to complete the meta-analysis. The meta-analysis showed that the Chinese medicine decoctions significantly reduced the oral ulcer score in the experimental group when compared with the control group (MD = −0.43, 95% CI [−0.70, −0.16], *P*=0.002; Figure 5(a)).

#### 3.5.2. Eye Lesion

Eleven RCTs [15, 16, 18–20, 22, 24, 25, 27, 29, 30] quantified eye lesions. Based on the result of the heterogeneity test (*P* < 0.00001, *I*^2^ = 80%), the random effects analysis model was used to complete the meta-analysis. The meta-analysis showed that the Chinese medicine decoctions significantly reduced the eye lesion score in the experimental group when compared with the control group (MD = −0.52, 95% CI [−0.74, −0.31], *P* < 0.00001; Figure 5(b)).

#### 3.5.3. Genital Ulcer

Eleven RCTs [15, 16, 18–20, 22, 24, 25, 27, 29, 30] quantified genital ulcers. Based on the result of heterogeneity test (*P*=0.002, *I*^2^ = 64%), the random effects analysis model was used to complete the meta-analysis. The meta-analysis showed that the Chinese medicine decoctions significantly reduced the genital ulcer score in the experimental group when compared with the control group (MD = −0.52, 95% CI [−0.77, −0.27], *P* < 0.0001; Figure 5(c)).

#### 3.5.4. Skin Lesion

**Figure 6:**
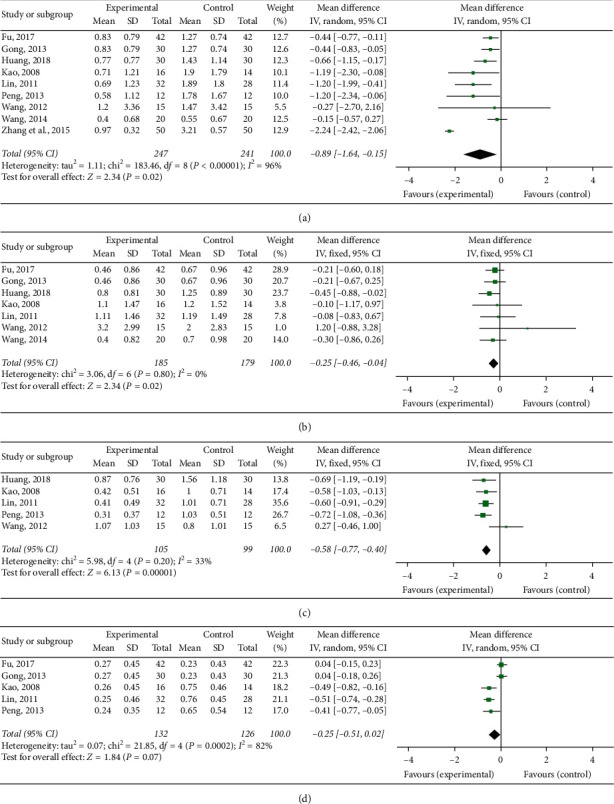
Forest plots of clinical features score: (a) skin lesion, (b) pathergy reaction, (c) arthropathy, and (d) fever.

Nine RCTs [15, 16, 18–20, 22, 25, 27, 29] quantified skin lesions. Based on the result of the heterogeneity test (*P* < 0.00001, *I*^2^ = 96%), the random effects analysis model was used to complete the meta-analysis. The meta-analysis showed that the Chinese medicine decoctions significantly reduced the skin lesion score in the experimental group when compared with the control group (MD = −0.89, 95% CI [−1.64, −0.15], *P*=0.02; Figure 6(a)).

#### 3.5.5. Pathergy Reaction

Seven RCTs [15, 16, 18–20, 25, 27] quantified pathergy reactions. Based on the result of the heterogeneity test (*P*=0.80, *I*^2^ = 0%), the fixed effect analysis model was used to complete the meta-analysis. The meta-analysis showed that the Chinese medicine decoctions significantly reduced the pathergy reaction score in the experimental group when compared with the control group (MD = −0.25, 95% CI [−0.46, −0.04], *P*=0.02; Figure 6(b)).

#### 3.5.6. Arthropathy

Five RCTs [18–20, 22, 25] quantified arthropathy. Based on the result of the heterogeneity test (*P*=0.20, *I*^2^ = 33%), the fixed effect analysis model was used to complete the meta-analysis. The meta-analysis showed that the Chinese medicine decoctions significantly reduced the arthropathy score in the experimental group when compared with the control group (MD = −0.58, 95% CI [−0.77, −0.40], *P* < 0.00001; Figure 6(c)).

#### 3.5.7. Fever

Five RCTs [15, 16, 19, 20, 22] quantified fever. Based on the result of the heterogeneity test (*P*=0.0002, *I*^2^ = 82%), the random effects analysis model was used to complete the meta-analysis. No significant difference in the fever score was observed between the experimental group and the control group (MD = −0.25, 95% CI [−0.51, 0.02], *P*=0.07; Figure 6(d)).

#### 3.5.8. CRP

**Figure 7:**
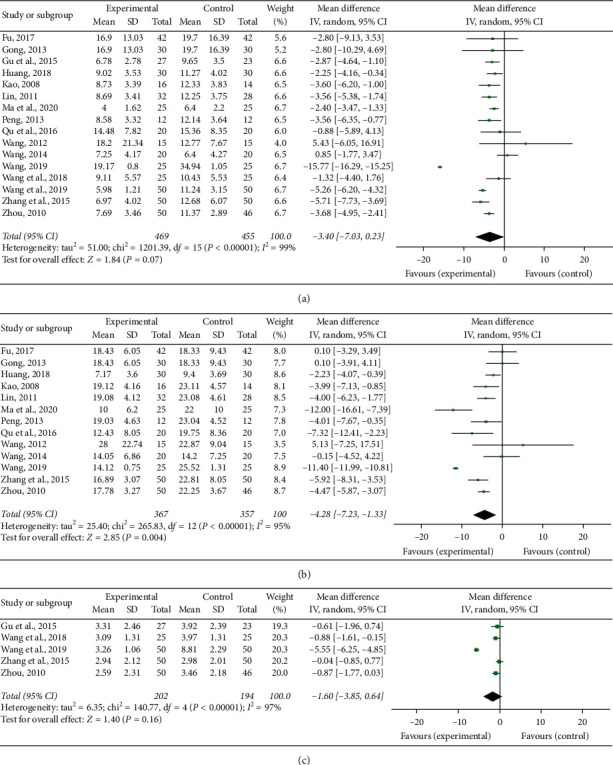
Forest plots of laboratory indexes level: (a) CRP, (b) ESR, and (c) IgA.

Sixteen RCTs [15–30] measured CRP. Based on the result of the heterogeneity test (*P* < 0.00001, *I*^2^ = 99%), the random effects analysis model was used to complete the meta-analysis. No significant difference in the CRP level was observed between the experimental group and the control group (MD = −3.40, 95% CI [−7.03, 0.23], *P*=0.07; Figure 7(a)).

#### 3.5.9. ESR

Thirteen RCTs [15, 16, 18–25, 27, 29, 30] measured ESR. Based on the result of the heterogeneity test (*P* < 0.00001, *I*^2^ = 95%), the random effects analysis model was used to complete the meta-analysis. The meta-analysis showed that the Chinese medicine decoctions decreased the ESR in the experimental group significantly more than the control group (MD = −4.28, 95% CI [−7.23, −1.33], *P*=0.004; Figure 7(b)).

#### 3.5.10. IgA

Five RCTs [17, 26, 28–30] measured IgA. Based on the result of the heterogeneity test (*P* < 0.00001, *I*^2^ = 97%), the random effects analysis model was used to complete the meta-analysis. No significant difference in the IgA level was observed between the experimental group and the control group (MD = −1.60, 95% CI [−3.85, 0.64], *P*=0.16; Figure 7(c)).

### 3.6. A Meta-Analysis of Modified Gancao Xiexin Decoction for BD Treatment

**Figure 8:**
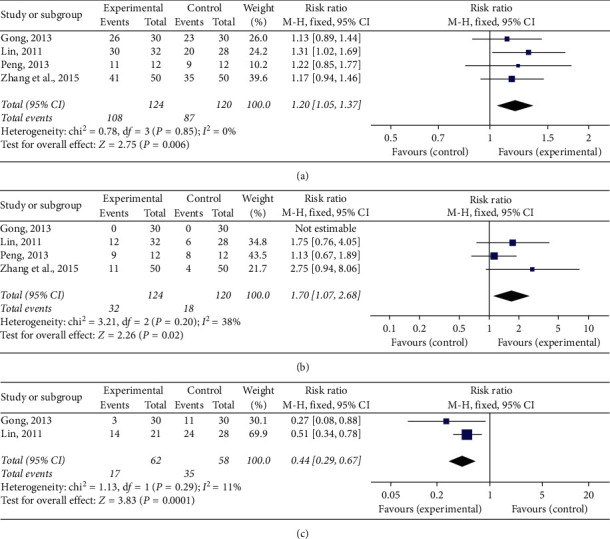
Forest plots of (a) total effective rate, (b) recovery rate, and (c) recurrence rate. (A meta-analysis of modified Gancao Xiexin Decoction for BD treatment.)

**Figure 9:**
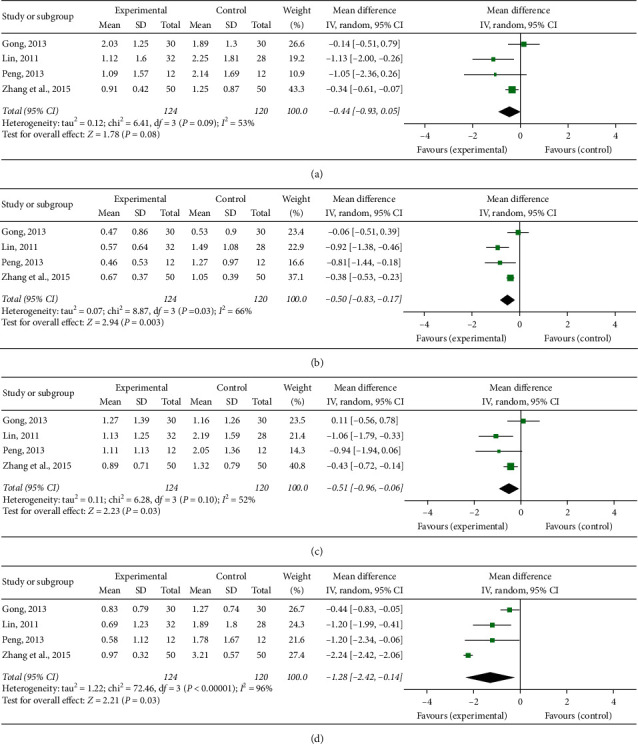
Forest plots of (a) oral ulcer, (b) eye lesion, (c) genital ulcer, and (d) skin lesion. (A meta-analysis of modified Gancao Xiexin Decoction for BD treatment.)

**Figure 10:**
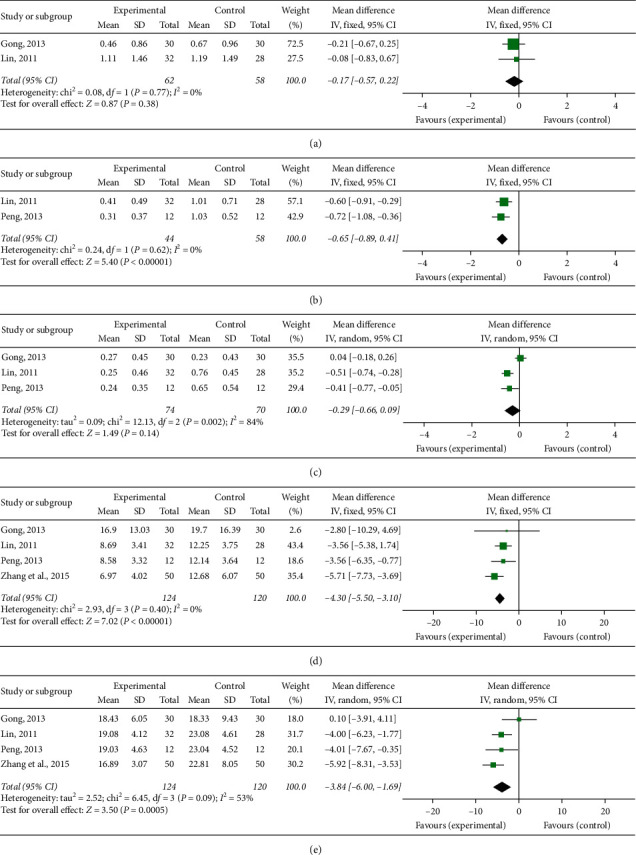
Forest plots of (a) pathergy reaction, (b) arthropathy, (c) fever, (d) CRP, and (e) ESR. (A meta-analysis of modified Gancao Xiexin Decoction for BD treatment.)

Among the 16 included RCTs, modified Gancao Xiexin decoction was used in five RCTs. In one of the five RCTs, some of the patients took other herbal decoctions instead of modified Gancao Xiexin decoction, and the exact number of patients taking modified Gancao Xiexin decoction was not available. A meta-analysis of the remaining four RCTs [16, 20, 22, 29] was conducted (see Figures 8–10). The results indicated that modified Gancao Xiexin decoction was effective for BD compared with control groups for all the primary outcomes, and for the secondary outcomes of eye lesions, genital ulcers, skin lesions, arthropathy, CRP, and ESR.

### 3.7. AEs

AEs were reported in 11 out of 16 RCTs. For eight RCTs [15, 16, 21, 23–25, 27, 28], the incidence of AEs in the experimental group was lower than in the control group. For one RCT [18], no AEs occurred in the experimental or control groups. For one RCT [17], the incidence of AEs in the control group was lower than that in the experimental group. For one RCT [30], diarrhea occurred in three patients in the experimental group, and no AEs occurred in the control group. Generally, the major AEs of Chinese medicine decoctions were gastrointestinal reactions, sleepiness, and dizziness; no severe kidney or liver damage was reported. Five RCTs did not mention AEs. Specific AEs are presented in Table 3.

### 3.8. Publication Bias

**Figure 11:**
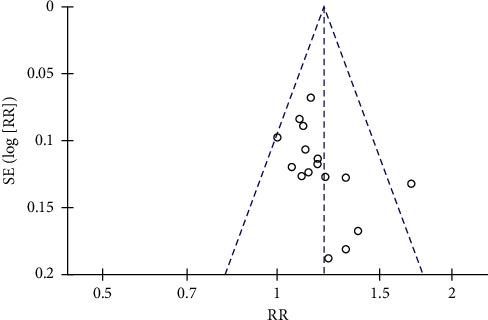
The funnel plot of the total effective rate.

**Figure 12:**
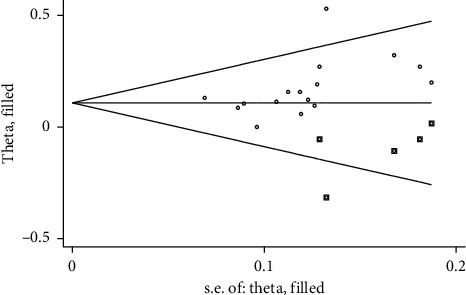
The filled funnel plot of the total effective rate.

A funnel plot was drawn for the total effective rate of 16 RCTs through the RevMan 5.4.1 software (see Figure 11). Visual inspection of the funnel plot showed asymmetry, suggesting a potential publication bias. For this reason, the Stata 14.0 software was used to conduct a sensitivity analysis by the trim and fill method [31]. The imputed studies formed a symmetrical funnel plot (see Figure 12). Without trim and fill, the combined effect sizes calculated by the fixed effect model and the random effects model were the same, RR = 1.157 (95% CI [1.095, 1.221], *P* < 0.001). With trim and fill, the combined effect size calculated by the fixed effect model was RR = 1.113 (95% CI [1.058, 1.171], *P* < 0.001) and the random effects model was RR = 1.114 (95% CI [1.043, 1.190], *P*=0.001). The RR values were similar, and the effects of intervention were both statistically significant before and after trim and fill. This indicated that publication bias had little effect on the conclusion.

## 4. Discussion

### 4.1. Summary of Main Results

The meta-analysis of the 16 RCTs indicates that Chinese medicine decoctions have, compared to controls, a significantly higher total effective rate and recovery rate and significantly lower recurrence rate, clinical feature scores (oral ulcers, eye lesions, genital ulcers, skin lesions, arthropathy, and pathergy reaction), and laboratory index levels (ESR). However, the evidence is inadequate to support that Chinese medicine decoctions are effective in lowering fever, and CRP and IgA levels. As for safety, in one RCT, diarrhea occurred in three patients after the use of Chinese medicine decoctions, which might be related to the gastrointestinal motility adjustment caused by herbs. In the other eight studies that reported adverse events, the incidence of adverse events in the experimental group was lower than in the control group, which indicated Chinese medicine decoctions may have lower incidences of adverse events than western drugs. In summary, Chinese medicine decoctions are valuable as a complementary and alternative therapy in the treatment of BD. The review covered a range of classical prescriptions, such as Gancao Xiexin decoction, Huanglian Wendan decoction, and Zhigancao decoction. Gancao Xiexin decoction and the high frequency herbs will be discussed from the perspective of individualized medicine and pharmacology. It may provide a reference for the exploration of more targeted treatment of BD with herbs.

### 4.2. Agreements and Disagreements with Another Relevant Review

Compared to the latest published meta-analysis on the treatment of BD with herbal medicine [32], 11 new RCTs were included in this review. For the composition of the experimental group, eight RCTs used only Chinese medicine decoctions, and the other eight RCTs used Chinese medicine decoctions combined with the same treatment as the control group. For the outcome measures, two new clinical features (fever and pathergy reaction) and one new laboratory index (IgA) were added to the analysis. From the results, it was concluded that Chinese medicine decoctions were effective and safe in the treatment of BD.

### 4.3. Discussion of Precision Medicine

Precision medicine is a medical model for prevention, diagnosis, and treatment that aims to achieve an optimal therapeutic regimen for an individual. Treatment based on TCM syndrome differentiation meets this requirement and is a good example of this concept. In the theory of TCM, a syndrome is composed of several subjective symptoms and objective signs, and individual differences in patients are taken into account. As in precision treatment, the most appropriate decoction is then sought for each individual. In TCM, there are several guiding principles by which syndromes are differentiated: the Zang-fu viscera syndrome differentiation, six-meridian syndrome differentiation, and eight-principle syndrome differentiation. It can be helpful to discuss them in the context of deficiency syndrome and sthenia syndrome (two syndromes based on eight-principle syndrome differentiation).

Damp-heat syndrome is one type of sthenia syndrome, commonly seen in patients with BD [33]. It is similar to the acute episodes of BD, in which ulcers present as red in color, swollen in form, and severe in pain, and may be accompanied by fever [34]. Damp-heat syndrome is closely related to immunity, inflammatory response, intestinal flora, and glucolipid metabolism [35]. In the decoctions in this review, Gancao Xiexin decoction includes a combination of herbs with Rhizoma Pinelliae, Rhizoma Zingiberis, Rhizoma Coptidis, and Radix Scutellariae for the treatment of damp-heat syndrome. Modified Gancao Xiexin decoction was used in five RCTs. The potential mechanism of action of Gancao Xiexin decoction is worth exploring and is discussed in Section 4.4.

Deficiency syndrome is opposite to sthenia syndrome and is related to immune dysfunction or decreased immune function [36]. The ulcers are light in color, flat or sunken in form, with healing difficulty and slightly painful [34]. The symptoms of BD are recurrent in a long course and thus require higher energy expenditure from the body. Astragalus membranaceus is an example of the tonic herbs and was applied in six RCTs. In fact, deficiency syndrome and sthenia syndrome do not exist in isolation, and the two can occur alternately in the same patient. A patient may even show deficiency in some aspects, but sthenia in others. Clinical treatment based on syndrome differentiation is therefore adjustable. The specific herbs that are effective against particular syndromes may in fact suggest more targeted exploration into possible pharmacological mechanisms.

### 4.4. Discussion of Pharmacology

Research on the potential mechanisms of the herbal drugs has been somewhat limited because of the unclear pathogenesis of BD. The anti-inflammatory and immunomodulatory effects of traditional Chinese medicine, as well as the effects of its promotion of ulcer healing, deserve more attention. Gastrointestinal manifestations of BD are important as they are closely associated with morbidity and mortality [37]. Although the ileocecum is the region most commonly involved, BD may affect any part of the digestive tract and a variety of gastrointestinal organs [38, 39]. Interestingly, Gancao Xiexin decoction is more widely used in digestive system diseases, such as oral ulcers, gastric ulcers, and ulcerative colitis. Gancao Xiexin decoction is a classic prescription recorded in Shang-Han-Lun (Treatise on Cold Pathogenic Diseases) and Jin-Kui-Yao-Lue (Synopsis of Golden Chamber). It has been inherited and developed in different clinical areas because of its classic herbal combination and considerable clinical effect. It has been shown to exert an antireflux esophagitis effect, antiulcerative colitis effect, antioral ulcer effect, and protective effect against liver damage. It has also been shown to regulate gastric mucus secretion, enhance immune function, and improve antihypoxia ability [40]. In clinical studies on treating recurrent aphthous ulcers, Gancao Xiexin decoction was found to regulate the imbalance of T lymphocyte subsets including the ratio of CD4^+^/CD8^+^ and the number of CD3^+^, CD4^+^, and CD8^+^ [41–43]. T cells are the main lymphocytes involved in the pathogenesis of BD, also have an activated phenotype, and can produce inflammatory cytokines [44, 45]. In clinical studies on treatments for ulcerative colitis and gastric ulcers, Gancao Xiexin decoction was proved to adjust the level of serum inflammatory factors including TNF-*α*, IFN-*γ*, IL-8, IL-12, IL-17, and IL-23 [46–48]. Among them, the IL-17/IL-23 pathway may play an important role in the mechanism of BD [49], and Th1 cell infiltrates including IL-8, IL-12, TNF-*α*, and IFN-*γ* were reported in gastrointestinal manifestations of BD [5]. Therefore, Gancao Xiexin decoction might be a potential treatment for the gastrointestinal lesions and recurrent aphthous ulcer lesions of BD.

Although other decoctions are not as frequently reported as Gancao Xiexin decoction, some key herbs are noteworthy. Radix Glycyrrhizae is the most widely used herb, appearing in almost every decoction. The active compounds isolated from Radix Glycyrrhizae have anti-inflammatory, antiviral, antimicrobial, antitumor, and immunoregulatory effects [50]. Among them, mainly glycyrrhizin mediates the anti-inflammatory activity of liquorice and promotes the healing of stomach and oral ulcers. Its anti-inflammatory effect is similar to mineralocorticoids and glucocorticoids [51]. Glucocorticoids are one class of drugs used to treat BD. This means that Chinese medicine may play a similar pharmacological role to western drugs, but with fewer adverse events.

Radix Astragali seu Hedysari, the most frequent tonic herb in the included studies, is considered to have immunoregulatory, anti-inflammatory, and antioxidative effects, as well as antiviral and antihyperglycemic activity [52]. A systematic review showed that Astragalus membranaceus may have an immunomodulatory effect on the Th17/Treg axis [53], and Th17 cells are considered to play an important part in BD pathogenesis [49].

Rhizoma Coptidis was used in eight RCTs. Berberine (BBR) is the abundant and main active ingredient of Rhizoma Coptidis [54] and widely used in the treatment and research of inflammatory diseases. Yang et al. reported that BBR may reduce STAT3 phosphorylation to inhibit the Th17 response in the patients with ocular BD [55]. Recently, Li et al. discovered that its natural oxoderivative, oxyberberine, has greater anti-inflammatory activity than BBR and a promising future in the area of inflammation [56].

In this review, Radix Angelicae Sinensis, Radix Scutellariae, Rhizoma Pinelliae (including Rhizoma Pinelliae Preparata), and Rhizoma Zingiberis (including Rhizoma Zingiberis Recens) were used in more than half of included RCTs. Their extracts have been demonstrated to have anti-inflammatory effects, which can suppress the expression of inflammatory cytokines such as TNF-*α* [57–60]. Interestingly, by comparing the phytochemicals and anti-inflammatory and antioxidant properties of sun-, freeze- and oven-dried ginger extracts, it was concluded that the drying process had a positive effect on ginger bioactivities, especially sun-dried ginger [61]. This reflects the superiority of traditional processing methods for herbs. In general, TNF-*α* is an important target of pharmacological action of herbs, and TNF blockade is an essential therapeutic progress for treating BD [9], suggesting that these herbs have the potential to be more targeted treatments for BD.

Gancao Xiexin decoction consists of Radix Glycyrrhizae, Rhizoma Coptidis, Radix Scutellariae, Rhizoma Pinelliae, Rhizoma Zingiberis, Radix Ginseng, and Fructus Jujubae. Radix Ginseng can exert anti-inflammatory and immunomodulatory effects, and the terpenoid saponins have some structural similarities to steroids [62]. Fructus Jujubae was considered protective of the gastrointestinal tract against intense inflammatory stimulation, alleviating inflammatory bowel disease [63], and played bidirectional immunoregulatory roles [64]. The other five ingredients of Gancao Xiexin have already been discussed above.

### 4.5. Limitations and Prospects

Although all the included studies were RCTs, some did not report their specific randomization methods. The blinding methods were also unsatisfactory. Lastly, solid conclusions cannot be drawn due to small sample sizes and poor methodological quality. Therefore, well-designed, multicenter, RCTs with large sample sizes are needed.

Differences in the types and dosages of herbs may also be a source of statistical heterogeneity. We did not find large numbers of clinical studies of similar classical prescriptions, although modified Gancao Xiexin decoction was used in five RCTs. For this reason, more research should be done on the herbs and decoctions of high frequency in treating BD and their pharmacological effect.

The criteria of TCM syndrome determination are not completely uniform. Different studies have developed different criteria for syndrome differentiation. Even for the same type of syndrome, subjective symptoms and objective signs may not be identical. This makes it difficult to carry out large-scale clinical studies on a single syndrome. Thus, an authoritative, standardized, and unified standard of TCM syndrome diagnosis is necessary for clinical research.

## 5. Conclusion

From this systematic review and meta-analysis, it was concluded that Chinese medicine decoctions had the potential to be effective and safe treatments for BD. However, more large-scale, well-designed, double-blinded RCTs are needed to further investigate the effectiveness and safety of Chinese medicine decoctions in the treatment to BD.

## Figures and Tables

**Table 1:** Characteristics of the included studies.

Author (year)	Grouping (blinding)	Sample size		Mean age (year)		Sex (male/female)		Mean disease course (year)	
		Experimental	Control	Experimental	Control	Experimental	Control	Experimental	Control
Fu (2017) [15]	Random allocation by SPSS21.0 statistical software	42	42	36.47 ± 9.22	36.33 ± 9.05	20/22	23/19	—	
Gong (2013) [16]	Random allocation by SPSS16.0 statistical software	30	30	36.47 ± 9.22	36.33 ± 9.05	17/13	14/16	—	
Gu et al. (2015) [17]	Random allocation	27	23	30.2 ± 2.0	29.6 ± 1.7	12/15	13/10	2.5 ± 0.3	2.3 ± 0.6
Huang (2018) [18]	Simple randomization	30	30	38.00 ± 12.38	41.26 ± 11.82	12/18	11/19	5.20 ± 2.57	4.80 ± 2.20
Kao (2008) [19]	Random allocation	16	14	30.2	29.8	6/10	5/9	7.6	8.1
Lin (2011) [20]	Random allocation	32	28	30.2	29.6	12/20	10/18	7.6	8.1
Ma et al. (2020) [21]	Random number table	25	25	27.3	27.9	12/13	11/14	—	
Peng (2013) [22]	Random allocation	12	12	37.5 ± 8.4		15/9		10.4 ± 1.2	
Qu et al. (2016) [23]	Block randomization	20	20	36.95 ± 9.36	37.34 ± 8.95	12/8	11/9	3.54 ± 1.62	3.78 ± 1.48
Wang (2012) [25]	Completely random principle; single blind	15	15	34.20 ± 9.92	40 ± 11.24	10/5	8/7	6.65 ± 5.72	8.52 ± 8.24
Wang (2014) [27]	Completely random principle; single blind	20	20	38.00 ± 13.20	38.55 ± 11.89	10/10	11/9	—	
Wang (2019) [24]	Random number table	25	25	39.12 ± 2.45	39.60 ± 2.44	16/9	10/15	7.52 ± 0.87	7.40 ± 0.81
Wang et al. (2018) [28]	Random number table	25	25	46.54 ± 13.37	45.68 ± 12.98	15/10	17/8	—	
Wang et al. (2019) [26]	Random number table	50	50	32.7 ± 7.3	32.2 ± 6.9	27/23	26/24	2.1 ± 0.6	2.2 ± 0.8
Zhang et al. (2015) [29]	Random number table	50	50	40.00 ± 9.21	36.20 ± 8.10	28/22	31/19	5.21 ± 4.32	3.65 ± 1.53
Zhou (2010) [30]	Random allocation	50	46	—		20/30	18/28	—	

**Table 2:** Interventions and treatment course in the included studies.

Author (year)	Intervention		Course (month)
	Experimental	Control	
Fu (2017) [15]	(i) Same treatment as the control group (ii) Modified Jiawei Zhigancao decoction (administered warm three times a day; total daily dose 200 ml)	(i) Thalidomide (50 mg, qn, po)	3
Gong (2013) [16]	(i) Same treatment as the control group (ii) Modified Gancao Xiexin decoction (administered twice a day; total daily dose 200 ml)	(i) Thalidomide (50 mg, qn, po)	3
Gu et al. (2015) [17]	(i) Same treatment as the control group (ii) Modified Huatan Quyu decoction (administered twice a day; total daily dose 400 ml)	(i) Azathioprine (100 mg, qd, po): the dose was reduced after the condition stabilized, reduced 5 mg every two weeks. The maintenance dose was 5 mg/d.	2
Huang (2018) [18]	(i) Same treatment as the control group (ii) Modified Huanglian Wendan decoction (administered warm once after breakfast and once after dinner; total daily dose 600 ml)	(i) Thalidomide (50 mg, tid, po) (ii) Celebrex (0.2 g, bid, po)	3
Kao (2008) [19]	(i) Yiqi Tuodu decoction (administered twice a day; total daily dose 400 ml)	(i) Prednisone (10 mg, bid, po): the dose was reduced after the condition stabilized, reduced 5 mg every two weeks. The maintenance dose was 5 mg/d.	2
Lin (2011) [20]	(i) Modified Gancao Xiexin decoction (administered twice a day; total daily dose 400 ml) (ii) Genital ulcers treated by Kushen decoction fuming-washing, 2-3 times a day	(i) Prednisone (10 mg, bid, po) (ii) Azathioprine (100 mg, qd) The dose was reduced after the condition stabilized, reduced 5 mg every two weeks. The maintenance dose was 5 mg/d.	2
Ma et al. (2020) [21]	(i) Same treatment as the control group (ii) Treatment based on syndrome differentiation (a) Damp-toxin syndrome: modified Wuwei Xiaodu decoction (b) Damp-heat syndrome: modified Gancao Xiexin decoction (c) Yin deficiency and heat inside syndrome: modified Baihe Dihuang decoction or Zhibai Dihuang decoction	(i) Thalidomide (50 mg/d, po): adjust the dose according to the situation (ii) Vitamin B1 (20 mg/d, po) (iii) Vitamin B2 (10 mg/d, po) (iv) Vitamin C (0.2 g/d, po) (v) Diclofenac sodium (when necessary)	3
Peng (2013) [22]	(i) Modified Gancao Xiexin decoction	(i) Prednisone (10 mg, bid, po) (ii) Azathioprine (100 mg, qd) The dosage was adjusted appropriately according to the condition, and the maintenance dose was 5 mg/d	4
Qu et al. (2016) [23]	(i) The same treatment as the control group (ii) Modified Yiqi Jiedu Quyu decoction (administered twice a day; total daily dose 200 ml)	(i) Thalidomide (50 mg/d, po)	3
Wang (2012) [25]	(i) Self-designed basic decoction (administered warm once after breakfast and once after dinner)	(i) Thalidomide (50 mg, hs, po)	2
Wang (2014) [27]	(i) Self-designed basic decoction based on promoting qi and resolving toxin (administered warm once after breakfast and once after dinner)	(i) Thalidomide (50 mg, hs, po) (ii) Compound Vitamin B Tablets (50 mg, tid, po)	2
Wang (2019) [24]	(i) Modified Jiawei Xiaoyao powder (apply it in the form of decoction, administered warm once after breakfast and once after dinner)	(i) Thalidomide (50 mg, bid, po)	2
Wang et al. (2018) [28]	(i) Same treatment as the control group (ii) Modified Huatan Quyu decoction (administered twice a day; total daily dose 400 ml)	(i) Basic drug treatment for disease not directly related to Behcet's syndrome (ii) Iguratimod (25 mg, bid, po): after the condition stabilized, the dose was reduced to (25 mg, qd, po)	2
Wang et al. (2019) [26]	(i) Same treatment as the control group (ii) Modified Huatan Quyu decoction (administered twice a day; total daily dose 800 ml)	(i) Azathioprine (the dose was tapered after high-dose treatment)	2
Zhang et al. (2015) [29]	(i) Modified Gancao Xiexin decoction and Sanhuang (administered warm once after breakfast and once after dinner)	(i) Prednisone (30 mg, bid, po), after 2-3 weeks, depending on the improvement of symptoms, the maintenance dose was gradually reduced to 10–20 mg/time (ii) Thalidomide (50 mg, hs, po)	3
Zhou (2010) [30]	(ii) Gan Chi decoction (administered warm twice a day; total daily dose 200 ml)	(i) Prednisone (10 mg/d, po)	3

**Table 3:** Outcome measures and adverse events in the included studies.

Author (year)	Primary outcomes	Secondary outcomes		Adverse events
		Clinical feature score	Laboratory index level	
Fu (2017) [15]	Total effective rate Recurrence rate (3 months)	Oral ulcer; eye lesion Genital ulcer; skin lesion Pathergy reaction; fever	ESR; CRP	Sleepiness, dizziness (experimental: 2; control: 5) Dry mouth, dry skin (experimental: 1; control: 4) Foreign body sensation on the skin (control: 1) Abnormal urinary occult blood (experimental: 1) Abnormal liver function (control: 2)
Gong (2013) [16]	Total effective rate Recurrence rate (3 months)	Oral ulcer; eye lesion Genital ulcer; skin lesion Pathergy reaction; fever	ESR; CRP	Sleepiness, dizziness (experimental: 1; control: 5) Dry mouth, dry skin (experimental: 1; control: 3) Foreign body sensation on the skin (control: 1)
Gu et al. (2015) [17]	Total effective rate Recovery rate	—	CRP; IgA	Skin rash (experimental: 1; control: 1) Hypoleucocytosis (experimental: 1; control: 1) peripheral sensory neuropathy (experimental: 2) Edema (experimental: 1) Constipation (experimental: 1) Sleepiness (experimental: 4) Inappetence (control: 2) Nausea and vomiting (control: 3) Dizziness, headache (control: 2)
Huang (2018) [18]	Total effective rate Recovery rate	Oral ulcer; eye lesion Genital ulcer; skin lesion Pathergy reaction; Arthropathy	ESR; CRP	No adverse events occurred
Kao (2008) [19]	Total effective rate Recovery rate Recurrence rate (4 months)	Oral ulcer; eye lesion Genital ulcer; skin lesion Pathergy reaction; Arthropathy Fever	ESR; CRP	—
Lin (2011) [20]	Total effective rate Recovery rate Recurrence rate (4 months)	Oral ulcer; eye lesion Genital ulcer; skin lesion Pathergy reaction; Arthropathy Fever	ESR; CRP	—
Ma et al. (2020) [21]	Total effective rate	—	ESR; CRP	Dizziness (experimental: 2; control: 3) Lower limb numbness (control: 1) Hypoleucocytosis (control: 1) Liver damage (experimental: 1; control: 2)
Peng (2013) [22]	Total effective rate Recovery rate	Oral ulcer; eye lesion Genital ulcer; skin lesion Arthropathy; fever	ESR; CRP	—
Qu et al. (2016) [23]	Total effective rate Recovery rate	—	ESR; CRP	Dizziness, sleepiness (experimental: 1; control: 2) Constipation (control: 3) Skin pruritus (control: 1) Scant menstrual flow (control: 1)
Wang (2012) [25]	Total effective rate Recovery rate Recurrence rate (3 months)	Oral ulcer; eye lesion Genital ulcer; skin lesion Pathergy reaction; arthropathy	ESR; CRP	Loose stool (experimental: 2) Sleepiness, nausea, dizziness, constipation (control: 1) More than one kind of adverse events (control: 10)
Wang (2014) [27]	Total effective rate Recovery rate Recurrence rate (3 months)	Oral ulcer; eye lesion Genital ulcer; skin lesion Pathergy reaction	ESR; CRP	Diarrhea (experimental: 1) Sleepiness (control: 3) Dry mouth, skin rash (control: 1) Constipation (control: 2)
Wang (2019) [24]	Total effective rate Recovery rate	Oral ulcer; eye lesion Genital ulcer	ESR; CRP	Slight abdominal distension (control: 5) slight constipation (control: 3)
Wang et al. (2018) [28]	Total effective rate Recovery rate	—	CRP; IgA	Hypoleucocytosis (experimental: 1; control: 2) Nausea (experimental: 2; control: 1) Stomach distension (experimental: 1) Inappetence (control: 1) Elevated aminotransferase (control: 1)
Wang et al. (2019) [26]	Total effective rate Recovery rate	—	CRP; IgA	—
Zhang et al. (2015) [29]	Total effective rate Recovery rate	Oral ulcer; eye lesion Genital ulcer; skin lesion	ESR; CRP; IgA	—
Zhou (2010) [30]	Total effective rate Recovery rate	Oral ulcer; eye lesion Genital ulcer	ESR; CRP; IgA	Diarrhea (experimental: 3)

## Data Availability

The data supporting this systematic review and meta-analysis are from previous studies and datasets, which have been cited. The processed data are available from the corresponding author upon reasonable request.

## Supplementary Materials

Supplementary Materials Table S1: retrieval strategy of the Cochrane database. Table S2: components of basic decoction and modification of prescriptions. Table S3: PRISMA checklist.
