# Supplementary material for: Effectiveness and Safety of Chinese Medicine Decoctions for Behcet's Disease: A Systematic Review and Meta-Analysis
Source: Evid Based Complement Alternat Med. 2021 Jul 17;2021:8202512. doi: 10.1155/2021/8202512 (PMC8313333; doi:10.1155/2021/8202512)
Supplement: Supplementary Materials — Table S1: retrieval strategy of the Cochrane database. Table S2: components of basic decoction and modification of prescriptions. Table S3: PRISMA checklist. [file 8202512.f1.zip › 8202512.f1/Table S1.docx]

| Table S1: Retrieval strategy of the Cochrane database | |
| --- | --- |
| ID | Search |
| #1 | MeSH descriptor: [Behcet Syndrome] explode all trees |
| #2 | ("BD" or "behcet disease" or "behcet's disease" or "behcet syndrome" or "behcet's syndrome"):ti,ab,kw (Word variations have been searched) |
| #3 | #1 or #2 |
| #4 | MeSH descriptor: [Medicine, Chinese Traditional] explode all trees |
| #5 | (TCM or herb or "herbal medicine" or "classical prescription" or "decoction"):ti,ab,kw (Word variations have been searched) |
| #6 | MeSH descriptor: [Medicine, Traditional] explode all trees |
| #7 | (Tang or San or Wan or Dan or Gao):ti,ab,kw (Word variations have been searched) |
| #8 | (Jian or Yin):ti,ab,kw (Word variations have been searched) |
| #9 | {OR #4-#8} |
| #10 | #3 and #9 |
