# Supplementary material for: Effectiveness and Safety of Chinese Medicine Decoctions for Behcet's Disease: A Systematic Review and Meta-Analysis
Source: Evid Based Complement Alternat Med. 2021 Jul 17;2021:8202512. doi: 10.1155/2021/8202512 (PMC8313333; doi:10.1155/2021/8202512)
Supplement: Supplementary Materials — Table S1: retrieval strategy of the Cochrane database. Table S2: components of basic decoction and modification of prescriptions. Table S3: PRISMA checklist. [file 8202512.f1.zip › 8202512.f1/Table S2.docx]

| **Table S2:** Components of basic decoction and modification of prescriptions | | | | | | | |  |  |
| --- | --- | --- | --- | --- | --- | --- | --- | --- | --- |
| Author (Year) | | basic decoction | | Components of basic decoction (daily dose) | | Modification of herbs and decoctions | |  |  |
|  |  |  |  |  |  |  |  |  | |
| Fu, 2017 | | modified Jiawei Zhigancao decoction | | Radix Glycyrrhizae (stir-frying) 15 g, Radix Glycyrrhizae 15 g, Radix Pseudostellariae 9 g, Radix Paeoniae Rubra 12 g, Radix Rehmanniae Recens 18 g, Semen Persicae 6 g, Flos Carthami 6 g, Radix Ophiopogonis 15 g, Fructus Forsythiae 9g, Radix Scrophulariae 15 g, Radix Scutellariae 9 g, Rhizoma Coptidis 9 g | | \ | |  | |
| Gong, 2013 | | modified Gancao Xiexin decoction | | Radix Glycyrrhizae (stir-frying) 9 g, Radix Glycyrrhizae 9 g, Radix Scutellariae 9 g, Rhizoma Coptidis 6 g, Radix Angelicae Sinensis 15 g, Radix Astragali seu Hedysari 18 g, Semen Coicis 30 g, Rhizoma Zingiberis 6 g, Radix Paeoniae Rubra 9 g, Cortex Moutan Radicis 9 g, Flos Lonicerae 20 g, Fructus Forsythiae 9 g, Pericarpium Citri Reticulatae 6 g | | • scant menses, amenorrhea, amnesia, insomnia: Fructus Ligustri Lucidi 20 g, Caulis Polygoni Multiflori 20 g • nausea, vomit, inappetenc: Rhizoma Atractylodis Macrocephalae 15 g, Fructus Amomi Villosi 6 g • dysphoria with feverish sensation in chest, palms and soles, tidal fever, hectic cheek: Rhizoma Anemarrhenae 12 g, Cortex Phellodendri 6 g • night sweating or bone steaming: Cortex Lycii 12 g | |  | |
| Gu et al, 2015 | | modified Huatan Quyu decoction | | Rhizoma Pinelliae 15 g, Radix Angelicae Sinensis 9 g, Radix Rehmanniae Recens 9 g, Poria 9 g, Semen Persicae (stir-frying) 12 g, Rhizoma Zingiberis Recens 25 g, Radix Paeoniae Rubra 9 g, Rhizoma Ligustici Chuanxiong 6 g, Pericarpium Citri Reticulatae 15 g, Radix Glycyrrhizae 30 g, Herba Commelinae 12 g, Carpesium Abrotanoides 15 g, Euonymus Alatus 12 g, Zaocys (processing with wine) 12 g | | • genital ulcer: Fructus Kochiae • anal ulcer: Fructus Sophorae (stir-frying) • eye lesion: Flos Buddlejae, Semen Cassiae • oral ulcer: Bingpeng powder (external application) • dampness-heat of liver channel: Radix Gentianae, Cortex Phellodendri, Semen Phaseoli | |  | |
|  | | | | | | | |  | |
| **Table S2:** (Continued) | | | | | | | |  |  |
| Author (Year) | | basic decoction | | Components of basic decoction (daily dose) | | Modification of herbs and decoctions | |  |  |
|  |  |  |  |  |  |  |  |  |  |
| Huang, 2018 | | modified Huanglian Wendan decoction | | Rhizoma Pinelliae 25 g, Rhizoma Coptidis 20 g, Radix Bupleuri 10 g, Pericarpium Citri Reticulatae15 g, Rhizoma Atractylodis Macrocephalae 20 g, Flos Lonicerae 15 g, Rhizoma Smilacis Glabrae 20 g, Semen Plantaginis 10 g, Cortex Moutan Radicis 15 g, Radix Glycyrrhizae 10 g | | \ | |  |  |
| Kao, 2008 | | Yiqi Tuodu decoction | | Radix Astragali seu Hedysari 45 g, Radix Paeoniae Alba 30 g, Folium Isatidis 15 g, Radix Glycyrrhizae 15 g, Radix Glycyrrhizae (stir-frying) 15 g, Tripterygium Wilfordii (decocted half an hour earlier) 9 g, Radix Angelicae Sinensis 9 g, Rhizoma Bletillae 6 g | | \ | |  |  |
| Lin, 2011 | | modified Gancao Xiexin decoction | | Radix Glycyrrhizae 30 g, Radix Scutellariae 25 g, Radix Ginseng 25 g, Rhizoma Zingiberis 25 g, Fructus Jujubae 15 g, Rhizoma Pinelliae 30 g | | • genital ulcer: Fructus Kochiae;  • anal ulcer: Fructus Sophorae (stir-frying); • eye lesion: Flos Buddlejae, Semen Cassiae; • oral ulcer: Bingpeng powder (external application);  • dampness-heat of liver channel: Radix Gentianae, Cortex Phellodendri, Semen Phaseoli | |  |  |
|  | | | | | | | |  |  |

| **Table S2:** (Continued) | | | | | | | |  |  |  |  |  |
| --- | --- | --- | --- | --- | --- | --- | --- | --- | --- | --- | --- | --- |
| Author (Year) | | basic decoction | | Components of basic decoction (daily dose) | | Modification of herbs and decoctions | |  |  |  |  |  |
|  |  |  |  |  |  |  |  |  | | | | |
| Ma et al, 2020 | | (a) modified Wuwei Xiaodu decoction (b) modified Gancao Xiexin decoction (c) modified Baihe Dihuang decoction or Zhibai Dihuang decoction | | (a) Flos Lonicerae 20 g, Flos Chrysanthemi Indici 20 g, Herba Violae 20 g, Cortex Moutan Radicis 15 g, Radix Gentianae 15 g, Radix Bupleuri 15 g, Rhizoma Smilacis Glabrae 20 g, Semen Coicis 20 g (b) Radix Glycyrrhizae 10 g, Radix Scutellariae 15 g, Radix Ginseng 10 g, Rhizoma Zingiberis 10 g, Rhizoma Coptidis 10 g, Rhizoma Pinelliae Preparata 20 g, Fructus Jujubae (c) Rhizoma Anemarrhenae 20 g, Cortex Phellodendri 15 g, Bulbus Lilii 20 g, Radix Rehmanniae Preparata 20 g, Rhizoma Smilacis Glabrae 20 g, Flos Chrysanthemi 20 g, Cortex Moutan Radicis 20 g | | • arthralgia: Caulis Spatholobi 20 g, Radix Gentianae Macrophyllae 20 g, Radix Achyranthis Bidentatae 20 g, Radix Stephaniae Tetrandrae 10 g, etc • erythema nodosa: Squama Manis 15 g, Semen Vaccariae 15 g, Radix Paeoniae Rubra 20 g, etc • conjunctival hyperemia, blurred vision: Semen Celosiae15 g, Flos Buddlejae 15 g, Fructus Gardeniae 20 g, etc • dry stool: Radix et Rhizoma Rhei 8 g, Cortex Magnoliae Officinalis 15 g, etc • insomnia: Caulis Polygoni Multiflori 20 g, Semen Ziziphi Spinosae (stir-frying) 20 g, etc | |  | | | | |
| Peng, 2013 | | modified Gancao Xiexin decoction | | Radix Glycyrrhizae 30 g, Rhizoma Pinelliae 15 g, Radix Scutellariae 10 g, Rhizoma Zingiberis 8 g, Radix Codonopsis 15 g, Rhizoma Coptidis 6 g, Fructus Jujubae | | • oral ulcer: Rhizoma Coptidis 5 g, Herba Lophatheri 10 g • genital ulcer: Radix Sophorae Flavescentis 15 g，Radix Gentianae 15 g, Cortex Phellodendri 10 g, Talcum 20 g • eye lesion: Rhizoma Coptidis 6 g, Flos Chrysanthemi Indici 10 g, Flos Eriocauli 10 g • dampness stagnancy due to spleen deficiency: Rhizoma Atractylodis 10 g, Cortex Phellodendri 10 g, Rhizoma Atractylodis Macrocephalae 10 g • blood stasis: Semen Persicae 10 g, Flos Carthami 10 g, Radix Salviae Miltiorrhizae 15 g | |  | | | | |
|  | | | | | | | |  | | | | |
| **Table S2:** (Continued) | | | | | | | |  |  |  |  |  |
| Author (Year) | | basic decoction | | Components of basic decoction (daily dose) | | Modification of herbs and decoctions | |  |  |  |  |  |
|  |  |  |  |  |  |  |  |  | | | |  |
| Qu et al, 2016 | | modified Yiqi Jiedu Quyu decoction | | Radix Astragali seu Hedysari 30 g, Radix Rehmanniae Recens 30 g, Rhizoma Curcumae 15 g, Radix Scutellariae 30 g, Rhizoma Smilacis Glabrae 30 g, Caragana sinica Rehd 30 g, Radix et Rhizoma Rhei (processed) 9 g, Radix Glycyrrhizae 12 g, Radix Glycyrrhizae (stir-frying) 12 g | | • erythema nodosa of lower limb: Radix Ranunculi Ternati 15 g, Fructus Forsythiae 12 g • genital ulcer: Caulis Sargentodoxae 30 g, Radix Sophorae Flavescentis 15 g • conjunctival hyperemia with pain: Radix Bupleuri 9 g, Fructus Gardeniae 9 g  • bitter taste in mouth, dysphoria: Rhizoma Coptidis 9 g, Herba Lophatheri 9 g  • dry mouth, low grade fever: Rhizoma Anemarrhenae 9 g, Cortex Phellodendri 9 g | |  | | | |  |
| Wang, 2012 | | self-designed basic decoction | | Radix Glycyrrhizae (stir-frying) 15 g, Radix Glycyrrhizae 15 g, Radix Astragali seu Hedysari 30 g, Folium Isatidis 15 g, Rhizoma Smilacis Glabrae 30 g, Fructus Forsythiae 12 g | | • syndrome of blazing fire-toxin, attack upward through the meridians: modified Longdan Xiegan decoction • syndrome of blazing fire-heat, toxin pervading both qi and nutrient phases: modified Liangying Qingqi decoction (Ding Ganren medical record) • syndrome of dampness-heat amassment, attack downward: modified Simiao Yongan decoction • syndrome of damp and hot cause the impairment of yin, deficiency fire with dampness: modified Zhibai Dihuang decoction • syndrome of blazing heat-toxin, bleeding due to blood heat: modified Xijiao Dihuang decoction | |  | | | |  |
|  | | | | | | | |  | | | |  |
| **Table S2:** (Continued) | | | | | | | |  |  |  |  |  |
| Author (Year) | | basic decoction | | Components of basic decoction (daily dose) | | Modification of herbs and decoctions | |  |  |  |  |  |
|  |  |  |  |  |  |  |  |  | | |  |  |
| Wang, 2014 | | self-designed basic decoction based on promoting qi and resolving toxin | | Radix Glycyrrhizae (stir-frying) 15 g, Radix Glycyrrhizae 15 g, Radix Astragali seu Hedysari 30 g, Tripterygium Wilfordii 12 g, Fructus Forsythiae 12 g, Folium Isatidis 15 g, Rhizoma Smilacis Glabrae 30 g, Radix Ampelopsis 12 g | | • high fever, polydipsia, oral ulcer (red in color, swollen in form): Gypsum Fibrosum 60 g, Rhizoma Anemarrhenae 30 g, Cornu Saigae Tataricae powder 1-2 g (administered after dissolved) • the ulcer is presenting red in color, swollen in form and severe in pain: Yushi Qingxin Liangge powder (Fructus Forsythiae 12 g, Radix Scutellariae 12 g, Fructus Gardeniae 12 g, Herba Menthae 9 g, Gypsum Fibrosum 30 g, Radix Platycodonis 12 g, Radix Glycyrrhizae 12 g, Herba Lophatheri 9 g) • arthralgia: Radix Angelicae Pubescentis 15 g, Rhizoma Ligustici Chuanxiong 15 g • conjunctival hyperemia with pain, constipation: Radix et Rhizoma Rhei • weakness (worsened after activity): Shengmai powder (Radix Pseudostellariae 9 g, Radix Ophiopogonis 9 g, Fructus Schisandrae Chinensis 6 g), Radix Astragali seu Hedysari • fever, extensive bleeding: Radix Lithospermi 15 g, Gypsum Fibrosum 30-90 g, or Zixue micropills (administered after dissolved) • abdominal pain, hematochezia: Radix Paeoniae Alba 30 g, Radix Glycyrrhizae12 g, Radix Sanguisorbae 15 g, Flos Sophorae 15 g | |  | | |  |  |
|  | | | | | | | |  | | |  |  |
| **Table S2:** (Continued) | | | | | | | |  |  |  |  |  |
| Author (Year) | | basic decoction | | Components of basic decoction (daily dose) | | Modification of herbs and decoctions | |  |  |  |  |  |
|  |  |  |  |  |  |  |  |  | |  |  |  |
| Wang, 2019 | | modified Jiawei Xiaoyao powder (apply it in the form of decoction) | | Radix Bupleuri 15 g, Radix Angelicae Sinensis 15 g, Radix Paeoniae Alba 15 g, Rhizoma Atractylodis Macrocephalae 15 g, Poria 15 g, Cortex Moutan Radicis 15 g, Fructus Gardeniae (stir-frying) 15 g, Rhizoma Cyperi 15 g, Fructus Aurantii 15 g, Cortex Magnoliae Officinalis 15 g, Rhizoma Zingiberis Recens 15 g, Herba Menthae 10 g, Radix Glycyrrhizae 10 g | | • conjunctival hyperemia, lacrimation, photophobia: Flos Sophorae 15 g, Flos Chrysanthemi 15 g, Herba Dendrobii 20 g • genital ulcer: Radix Sophorae Flavescentis 10 g, Herba Plantaginis 20 g, Cortex Phellodendri 10 g • erythema nodosa: Cortex Moutan Radicis 15 g, Herba Violae 10 g, Herba Taraxaci 20 g • arthralgia: Rhizoma Dioscoreae Tokoro 20 g, Herba Siegesbeckiae 30 g | |  | |  |  |  |
| Wang et al, 2018 | | modified Huatan Quyu decoction | | Rhizoma Pinelliae Preparata 15 g, Radix Angelicae Sinensis 9 g, Radix Rehmanniae Recens 9 g, Poria 9 g, Semen Persicae (stir-frying) 12 g, Radix Paeoniae Rubra 9 g, Rhizoma Ligustici Chuanxiong 6 g, Pericarpium Citri Reticulatae 15 g, Radix Glycyrrhizae 30 g, Herba Commelinae 12 g, Xiaojinpian 10 g, Radix Sophorae Tonkinensis 15 g, Carpesium Abrotanoides 15 g, Radix Tinosporae 10 g, Terminalia chebula Retz. 9 g, Rhizoma Sparganii 10 g, Rhizoma Curcumae 10 g | | • genital ulcer: Fructus Kochiae • anal ulcer: Fructus Sophorae (stir-frying) • eye lesion: Flos Buddlejae, Semen Cassiae • oral ulcer: Bingpeng powder (external application)  • dampness-heat of liver channel: Radix Gentianae, Cortex Phellodendri, Semen Phaseoli | |  | |  |  |  |
|  | | | | | | | |  | |  |  |  |
| **Table S2:** (Continued) | | | | | | | |  |  |  |  |  |
| Author (Year) | | basic decoction | | Components of basic decoction (daily dose) | | Modification of herbs and decoctions | |  |  |  |  |  |
|  |  |  |  |  |  |  |  |  |  |  |  |  |
| Wang et al, 2019 | | modified Huatan Quyu decoction | | Radix Angelicae Sinensis 9 g, Radix Rehmanniae Recens 9 g, Radix Paeoniae Rubra 9 g, Poria 9 g, Semen Persicae (stir-frying) 12 g, Herba Commelinae 12 g, Euonymus Alatus 12 g, Zaocys (processing with wine) 12 g, Carpesium Abrotanoides 15 g, Pericarpium Citri Reticulatae 15 g, Rhizoma Pinelliae 15 g, Rhizoma Ligustici Chuanxiong 6 g, Rhizoma Zingiberis Recens 25 g, Radix Glycyrrhizae 30 g | | • genital ulcer: Fructus Kochiae • anal ulcer: Fructus Sophorae (stir-frying) • eye lesion: Flos Buddlejae, Semen Cassiae • oral ulcer: Bingpeng powder | |  |  |  |  |  |
| Zhang et al, 2015 | | modified Gancao Xiexin decoction and Sanhuang | | Radix Glycyrrhizae 10 g, Radix Scutellariae 10 g, Radix Codonopsis 30 g, Rhizoma Zingiberis 10 g, Rhizoma Coptidis 6 g, Rhizoma Pinelliae Preparata 10 g, Radix Astragali seu Hedysari 30 g, Radix Angelicae Sinensis 20 g, Radix Paeoniae Rubra 30 g, Semen Phaseoli 30 g, Rhizoma Cimicifugae 10 g, Fructus Jujubae | | • acute episode: increase the dose of Radix Scutellariae and Rhizoma Coptidis • remission: Radix Astragali seu Hedysari | |  |  |  |  |  |
| Zhou, 2010 | | Gan Chi decoction | | Radix Scutellariae 9 g, Rhizoma Coptidis 6 g, Rhizoma Pinelliae Preparata 9 g, Rhizoma Zingiberis 9 g, Semen Phaseoli 18 g, Radix Angelicae Sinensis 12 g, Rhizoma Atractylodis Macrocephalae 9 g, Poria 12 g, Semen Coicis 15 g, Radix Rehmanniae Recens 12 g, Gypsum Fibrosum 18 g, Rhizoma Cimicifugae 9 g, Radix Glycyrrhizae 9 g | | \ | |  |  |  |  |  |
